# The Effect of Empowerment and Educational Programs on the Quality of Life in Iranian Women with HIV

**DOI:** 10.1177/2325958218759681

**Published:** 2018-03-22

**Authors:** Zahra Behboodi Moghadam, Elham Rezaei, Bahareh Sharifi, Saharnaz Nejat, Sara Esmaelzadeh Saeieh, Maryam Ordibeheshti Khiaban

**Affiliations:** 1 Associate Professor of Reproductive Health, Faculty of Nursing and midwifery, Tehran University of Medical Sciences, Tehran, Iran; 2 PhD student of Reproductive Health, Faculty of Nursing and midwifery, Tehran University of Medical Sciences, Tehran, Iran; 3 M.Sc. in Midwifery, Faculty of Nursing and midwifery, Tehran University of Medical Sciences, Tehran, Iran; 4 Professor in Epidemiology, School of Public Health, Tehran University of Medical Sciences, Tehran, Iran; 5 Assistant Professor of Reproductive Health, Social Determinants of Health research Center, Alborz University of Medical Sciences, Karaj, Iran; 6 Assistant Professor of Reproductive Health, Department of Midwifery, Tabriz branch, Islamic Azad University, Tabriz, Iran

**Keywords:** empowerment program, education program, quality of life, HIV positive women

## Abstract

AIDS affects physical, mental, social, and psychological health status. One of the goals of Health for All in the 21st century is to improve the quality of life. This study is a randomized clinical trial conducted on 120 HIV-positive women. Women were administered assessment questionnaires to be completed during the structured interview. After sample collection, participants were divided randomly into 3 groups by using the table of random numbers, then, respectively, received educational intervention, empowerment program, and routine procedures offered by the center and were followed by refilling the questionnaires 12 weeks after intervention. Depending on the type of data, chi-square, analysis of variance, and paired *t* test were used, and SPSS version 16 was used for data analysis. The finding showed that knowledge increased after intervention in educational (*P* = .02) and empowerment groups (*P* = .006); also empowerment group indicated significant difference in psychological (*P* = .006) and spiritual (*P* = .001) domains and their total quality of life (*P* = .004). According to this study, exposing HIV-positive women to empowerment education is effective in improving their quality of life.

## Introduction

It is believed that the millennium development goals cannot be achieved until the access to sexual and reproductive health-care services and prevention, treatment, care, and support of patients with HIV/AIDS become equally available to everyone.^1^ Human immunodeficiency virus (HIV) and acquired immune deficiency syndrome (AIDS) are the major health-care challenges over the world and the global epidemic. Over 90% of these people live in developing countries, and AIDS is considered one of the most common diseases in the today world. Thirty-seven million people are infected with HIV and 50 of whom are women. It is estimated that 2½ million children under the age of 15 years are living with HIV or AIDS.^2-4^ Diagnosis of HIV started late in the Iranian population but its growth rate was fast, that is, approximately 70 000 (47 000-110 000) individuals have been diagnosed with HIV of whom 19 000 (12 000-32 000) people comprised women and those under the age of 15.^5^ Majority of the patients with HIV are 25 to 34 years old. In addition, it is estimated that 70% of transmission are through injection drug users. The rest of them are infected through sexual intercourse, blood products, and mother-to-child infection transmission.^6^ In Iran, almost 69.8% of the infected people are drug addicts.^7^

The Middle East and North Africa (MENA) region has the lowest HIV prevalence rates (0.1%) in the world. This is in contrast to sub-Saharan Africa, which has the highest HIV prevalence rate (7.1%) of all regions. In the Middle East and North Africa, HIV and AIDS (38% increase in past 10 years, contrast to other regions where incident rates are declining) is increasingly becoming a concern. In Afghanistan, Pakistan, and Iran, injecting drug use is the major route of HIV transmission, which accounted for an estimated 90% of HIV cases in Libya. This being common in Oman and Bahrain, and a growing issue in Morocco and Egypt.^8^

The society’s negative attitude and social stigma toward HIV/AIDS not only affect patients’ physical and mental health but also cause many problems in their daily activities. HIV/AIDS affects every aspect of patients’ life, decreases their self-esteem, and increases their feeling of vulnerability. Moreover, it can cause physical symptoms, daily and social dysfunction, and confused thoughts. Furthermore, repeated doctor visits, the high-cost treatment, and complications caused by drugs reduce quality of life (QOL) in patients.^9^

Patients diagnosed with HIV/AIDS face lot of stigma in the society. They have disturbances in their social and interpersonal relationships and encounter problems in their sexuality, with a lack of social and financial support. Consequently, their mental and physical well-being, welfare, and QOL are jeopardized. Women are recognized as the most vulnerable group among patients with HIV/AIDS. The causes of women’ vulnerability are attributed to unequal opportunities provided to women in terms of health maintenance, promotion, and prevention. In general, the World Health Organization (WHO) defines the QOL as the individual’s judgment of his or her position in life according to the culture and values in which he or she lives, with the consideration of his or her objectives, expectations, and concerns. This multifactor concept consists of physical, psychological, social, environmental, and spiritual domains. The main idea behind the term QOL is the promotion of health with a focus on meeting the fundamental, material, and spiritual needs of the human being.^10,11^

One of the goals of the Health for All in the 21st century is to improvement the QOL and empowering individuals by enhancing their own QOL. Empowerment is defined as the expansion of people’s confidence, awareness, and decision-making skills to enhance health and health-care and improve their QOL. The key component of empowerment is the participation of people to help themselves and have a sense of control and power to achieve the empowered goals.^12^ One of the empowerment programs is an empowerment model designed based on the following 4 steps: (1) understanding the threat (2) problem solving (3) educational participation, and (4) evaluation.^13^

Women infected with HIV/AIDS are more vulnerable than men. Often women are deprived of social support, lose their family relationships, and experience financial problems. All these affect the QOL of HIV-positive women. Therefore, the reduction in women’s QOL seems unavoidable. The aim of this study was to investigate the effect of empowerment and educational programs on the QOL of women with HIV, given the role of midwives in improving the health of women, particularly those with HIV/AIDS.

## Methodology

This study was a randomized clinical trial conducted from June to the end of November 2012. Written informed consent was obtained from 120 women with HIV referred to the behavioral diseases consultation center of Imam Khomeini Hospital, Tehran, Iran. Inclusion criteria for the recruitment of the samples were as follows: literate, Iranian national, married, not taking addictive drugs, diagnosed with HIV in the last 5 years, aged 15 to 45 years, and having no chronic disease, malignancy, and psychiatric diseases.

The sociodemographic characteristics questionnaire was used to gather data about the samples’ age, educational level, occupation, marital status, husband’s educational level and occupation, husband’s infection status, use of contraception, socioeconomic status, how they got infected with HIV, and disease’s stage. Also, the knowledge assessment questionnaire and QOL questionnaire for patients with HIV World Health Organization quality of life in HIV infection, abbreviated version (WHOQOL-HIV-Bref) were the other data collection tools used in this study. The knowledge assessment questionnaire measured the awareness level in women and knowledge about AIDS. It consisted of 10 multiple-choice questions relating to issues such as the cause of AIDS, transmission routes of AIDS, and the most common route of AIDS’s transmission in the country. The scores of this questionnaire were based on the number of correct answers given to the questions, with 0 for wrong answer and 1 for correct answer. Therefore, the scores ranged from 0 to 10.

The HIV brief version (WHOQOL-HIV BREF) of the WHO QOL questionnaire consisted of 31 questions measuring the health status and QOL in 6 domains such as physical, psychological, environmental, spiritual health, the level of autonomy, and social relations. A 5-point Likert-type scale was used, with scores ranging from 1 to 5, in which 1 being the lowest score and 5 being the highest score for each question. The total range score of this questionnaire was 31 to 155, with a higher score indicating the better QOL. The WHOQOL-HIV BREF was prepared and designed in 2004 under the supervision of the WHO and was used in different countries. The translation and validation of this questionnaire was performed in 2009 in Malaysia. The reliability of this questionnaire was calculated using the Cronbach α coefficient, which was reported as 0.70 to 0.83. The validity and reliability of this questionnaire were determined by studies in other countries and Iran.^14-18^ For determining the validity of this questionnaire, 12 faculty members of Tehran University of Medical Sciences and Behavioral Medicine Counseling Center of Imam Khomeini Hospital were asked to give their feedbacks and suggestions. The questionnaire was filled by 20 women with HIV who had the inclusion criteria and were not included in the samples to determine its reliability through the calculation of Cronbach α coefficient, which was 0.78 to 0.87.

The convenience sampling method was used to recruit the samples. Before interventions, the questionnaires were filled via conducting the individual structured interviews with the samples in a quiet room away from any sort of disruption. Next, the samples were randomly divided into 3 groups by using the table of random numbers: the educational intervention group, empowerment intervention group, and control group.

The control group received only routine interventions delivered by nursing staff in the health-care center. The educational intervention group received education in two 60-minute sessions with 1-week interval in the form of lectures, discussions, and questions and answers. At the end of the second session, they were given an educational booklet containing educational materials.

In the empowerment intervention group, the empowerment program was presented in 4 steps as follows: understanding the threat, problem solving, educational participation, and evaluation. For increasing the level of perceived threat, the samples needed an expansion of their knowledge, cognition, and awareness about the nature of HIV/AIDS. Their knowledge was increased through education sessions and distributing educational pamphlets. Problem-solving session were held in the form of group discussions by a researcher and consultant psychologist. With regard to participation, educational booklets were handed over to the women at the end of fourth session to transfer the required knowledge to their family members.^14,19,20^ Evaluation was conducted in 2 sections, namely, the process of evaluation and summative evaluation. Process evaluation was assessed during meetings for reviewing problems in previous sessions and the women’s participation in the meetings. Twelve weeks after the intervention, the final evaluation was conducted by filling the questionnaires. Twelve weeks after the end of the educational sessions, the educational intervention and control groups filled the questionnaires. The collected data were analyzed using descriptive and inferential statistics via the SPSS software version 16. The chi-square test, analysis of variance, and paired *t* test were used to compare the groups.

The ethics committee affiliated with Tehran University of Medical Sciences, Tehran, Iran, approved this study’s research proposal and corroborated its ethical considerations (Date approved 2012-05-20. Reference number 2849/130/190). The participants were informed of the aim and process of the study and gave their informed consent for participating in this study.

## Results

A number of underlying and confounding factors affect the QOL. Therefore, 3 groups of HIV-positive women participated in this study. Before the intervention, the scores obtained by the 3 groups in the knowledge assessment questionnaire and the QOL questionnaire were compared, which showed no statistical significant differences. Therefore, the groups were homogeneous. According to Table 1, the women in the groups had no differences in terms of the sociodemographic characteristics.

**Table 1.** The Sociodemographic Characteristics of the Women in the Groups.

Groups	Empowerment Intervention		Control		Educational Intervention		*P* Value^a^
	n (%)	Mean (SD)	n (%)	Mean (SD)	n (%)	Mean (SD)	
Age							
20-24	5 (12.5)	33.38 (7.07)	7 (17.5)	32.28 (7.42)	2 (5)	33.68 (7)	.623
25-29	10 (25)		9 (22.5)		13 (32.5)		
30-34	9 (22.5)		8 (20)		7 (17.5)		
35-39	7 (17.5)		9 (22.5)		9 (22.5)		
>40	9 (22.5)		7 (17.5)		9 (22.5)		
Educational level							
Primary	6 (15)	-	7 (17.5)		11 (27.5)	-	.356
Secondary	12 (30)		11 (27.5)		8 (20)		
Diploma	15 (37.5)		14 (35)		14 (35)		
Academic	7 (17.5)		8 (20)		7 (17.5)		
Occupation**/**job							
Housewife	30 (75)	-	34 (85)	-	32 (80)	-	.165
Employee	10 (25)		10 (25)		8 (20)		
Socioeconomic status							
Good	6 (15)	-	12 (30)	-	13 (32.5)	-	.265
Moderate	19 (47.5)		5 (12.5)		18 (45)		
Bad	15 (37.5)		23 (57.5)		9 (22.5)		
No	16 (40)		6 (15)		11 (27.5)		
Husband’s educational level							
** **Primary	6 (15)	-	10 (25)	-	4 (10)	-	.412
** **Secondary	12 (30)		12 (30)		13 (32.5)		
** **Diploma	17 (42.5)		13 (32.5)		19 (47.5)		
** **Academic	5 (12.5)		5 (12.5)		4 (10)		
Husband’s occupation							
** **Unemployed	6 (15)	-	6 (15)	-	8 (20)	-	.194
** **Worker	12 (30)		5 (12.5)		5 (12.5)		
** **Employee	7 (17.5)		10 (25)		9 (22.5)		
** **Free business	15 (37.5)		19 (47.5)		18 (45)		
Husband’s infection status							
** **Yes	28 (70)	-	28 (70)	-	27 (67.5)	-	.652
** **No	9 (25.5)		10 (25)		10 (25)		
** **Don’t know	3 (7.5)		2 (5)		3 (7.5)		
Use of contraception							
** **Yes	35 (87.5)	-	27 (67.5)	-	32 (80)	-	.631
** **No	5 (12.5)		13 (32.5)		8 (20)		
How infected with HIV							
Sexual intercourse	23 (57.5)	-	30 (75)	-	28 (70)	-	.165
Needle contamination	3 (7.5)		1 (2.5)		3 (7.5)		
Blood transfusion	1 (2.5)		3 (7.5)		3 (7.5)		
Other	13 (32.5)		6 (15)		6 (15)		
Disease stage							
** **Asymptomatic	38 (95)	-	36 (90)	-	37 (92)	-	.659
** **Symptomatic	2 (5)		4 (10)		3 (7.5)		
** **Total	40 (100)		40 (100)		40 (100)		

Abbreviation: SD, standard deviation.

^a^ The *P* values were tested using the analysis of variance (ANOVA) and chi-square tests.

The scores of knowledge assessment showed statistically significant differences before and after the intervention between the educational intervention (*P* = .02) and empowerment intervention (*P* = .006) groups and also between the 3 groups (*P* = .002; Table 2). The QOL domain in the 3 groups showed statistically significant differences in psychological and spiritual domains and in the total QOL. Also, statistically significant differences were demonstrated in these dimensions between the groups before and after the intervention (Table 3, Figures 2–8).

**Table 2.** The Comparison of the Scores of the Knowledge Assessment Questionnaire in the Groups.

Groups	Before the Intervention	After the Intervention (12 weeks)	*P* Value (in Each Group) Paired *t* Test	*P* Value (between the Groups) ANOVA
	M (SD)	M (SD)		
Educational intervention	5.62 (1.86)	6.65 (2.62)	.028	.002
Empowerment intervention	5.98 (1.56)	7.58 (2.32)	.006	
Control	5.48 (1.79)	5.62 (1.82)	.291	

Abbreviations: ANOVA, analysis of variance; SD, standard deviation.

**Table 3.** Comparison of Quality of Life in HIV-Positive Women in 3 Groups.

Quality of Life	Groups	Previous Intervention	After Intervention (12 weeks)	*P* Value (in Each Group) Paired *t* Test	*P* Value^a^ (between Groups) ANOVA
		M (SD)	M (SD)		
Physical domain	Education	11.46 (2.57)	11.68 (2.55)	.216	.258
	Empowerment	11.75 (2.59)	11.79 (2.59)	.541	
	Control	12.41 (2.67)	12.23 (2.55)	.619	
Psychological domain	Education	12.08 (2.54)	12.11 (2.39)	.111	**.012**
	Empowerment	12.33 (2.77)	13.31 (2.94)	**.006**	
	Control	12.3 (2.21)	12.06 (2.16)	.265	
Social relationships	Education	11.06 (2.46)	11.21 (2.65)	.412	.610
	Empowerment	11.43 (2.95)	11.56 (2.32)	.612	
	Control	11.16 (2.31)	11.02 (2.62)	.512	
Environmental domain	Education	21.07 (4.6)	21.9 (4.5)	.982	.210
	Empowerment	21.32 (4.52)	21.27 (4.53)	.215	
	Control	20.55 (4.08)	21.65 (4.89)	.910	
Spiritual domain	Education	12.03 (2.5)	12.03 (2.20)	.167	**.015**
	Empowerment	12.5 (2.36)	14.01 (3.25)	**.001**	
	Control	12.07 (2.71)	12.1 (2.17)	.612	
Level of autonomy	Education	12.06 (2.06)	12.45 (2.38)	.641	.169
	Empowerment	12.53 (2.46)	12.57 (2.53)	.123	
	Control	12.83 (3.49)	12.94 (3.50)	.555	
Total quality of life	Education	91.07 (16.71)	91.75 (15.25)	.365	**.037**
	Empowerment	91.99 (15.64)	97.98 (16.64)	**.004**	
	Control	90.65 (15.87)	91.05 (15.5)	.241	

Abbreviations: ANOVA, analysis of variance; M, mean; SD, standard deviation.

**Figure 1.**
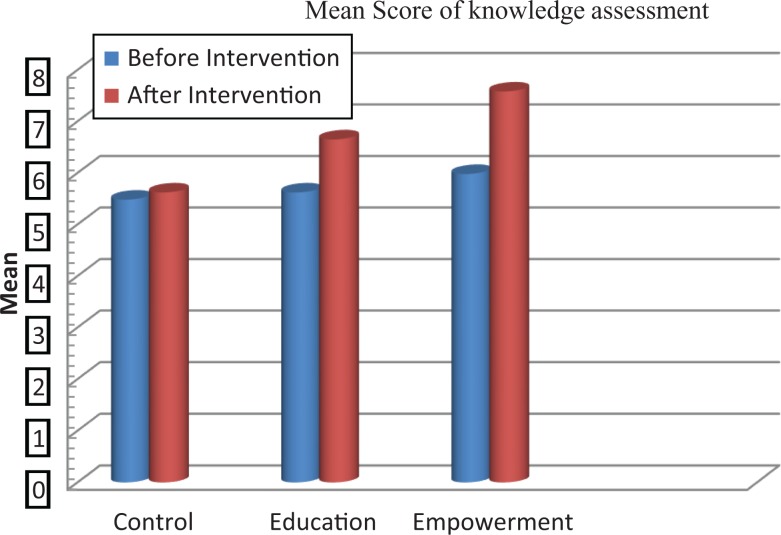
The mean score comparison of knowledge assessment questionnaire before and after intervention in 3 groups.

**Figure 2.**
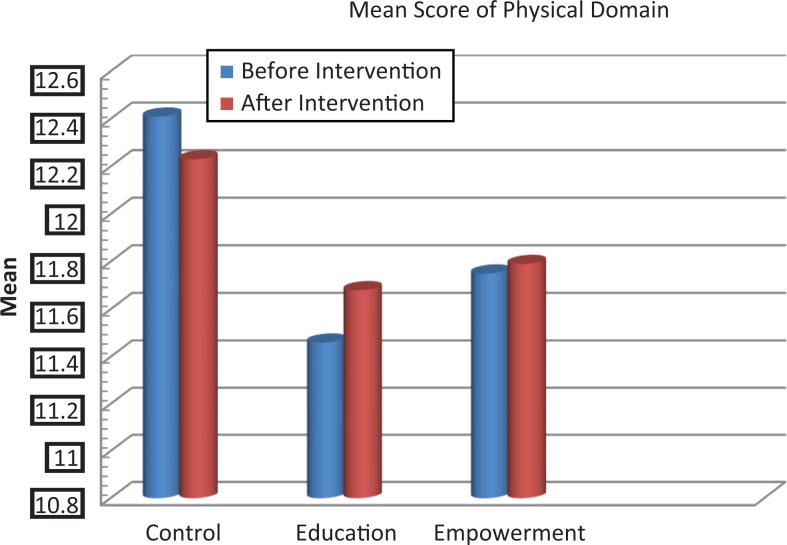
The mean score comparison of physical domain (quality of life) before and after intervention in 3 groups.

**Figure 3.**
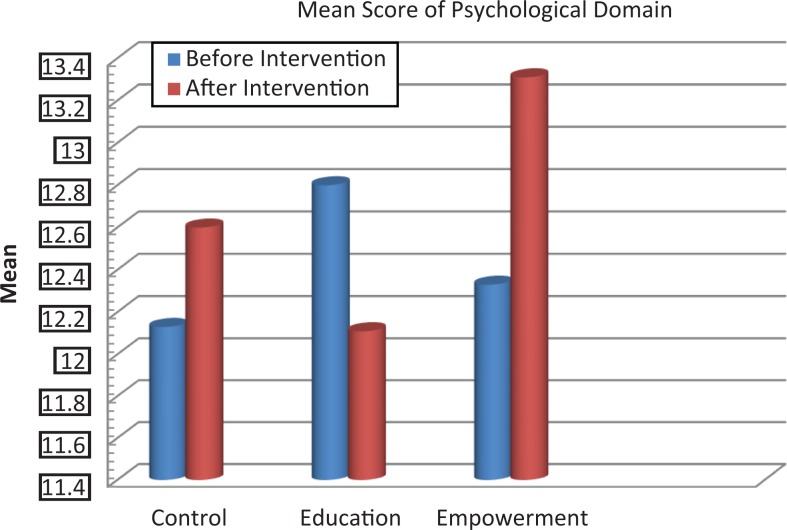
The mean score comparison of psychological domain (quality of life) before and after intervention in 3 groups.

**Figure 4.**
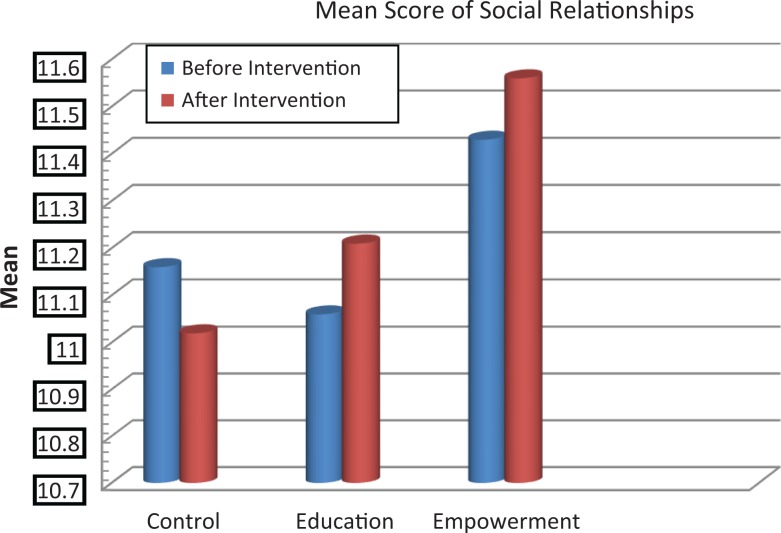
The mean score comparison of social relationships (domain of quality of life) before and after intervention in 3 groups.

**Figure 5.**
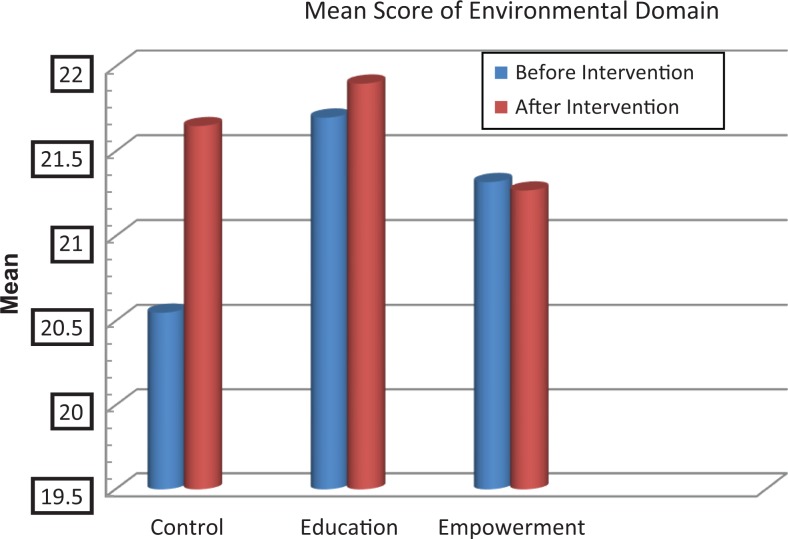
The mean score comparison of environmental domain (domain of quality of life) before and after intervention in 3 groups.

**Figure 6.**
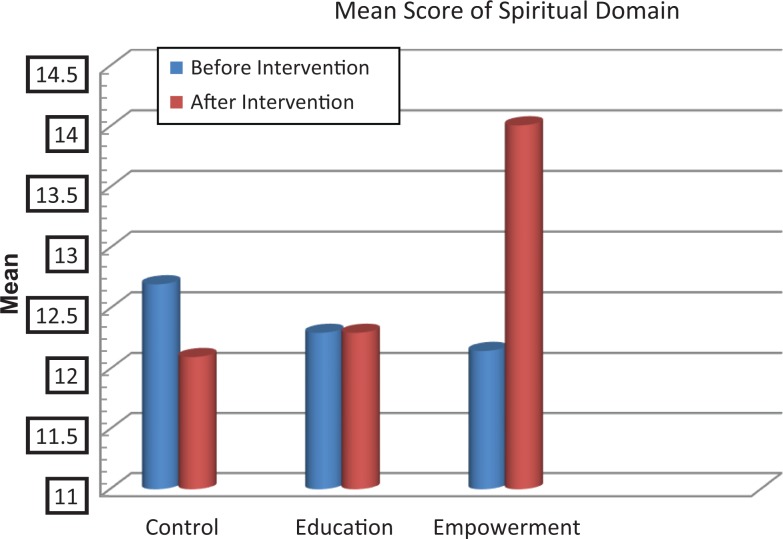
The mean score comparison of spiritual domain (quality of life) before and after intervention in 3 groups.

**Figure 7.**
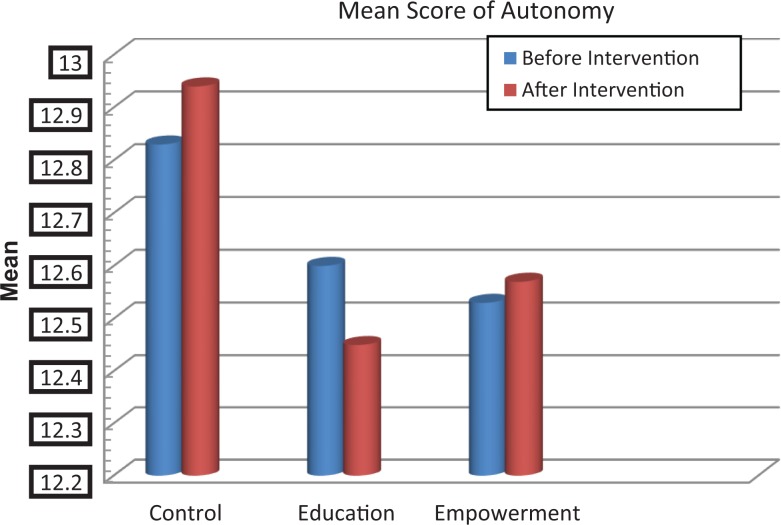
The mean score comparison of autonomy (domain of quality of life) before and after intervention in 3 groups.

**Figure 8.**
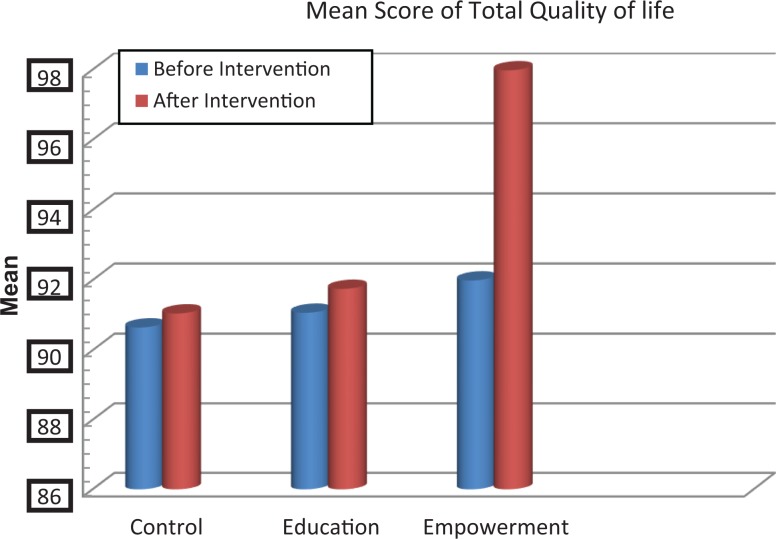
The mean score comparison of total quality of life before and after intervention in 3 groups.

**Figure 9.**
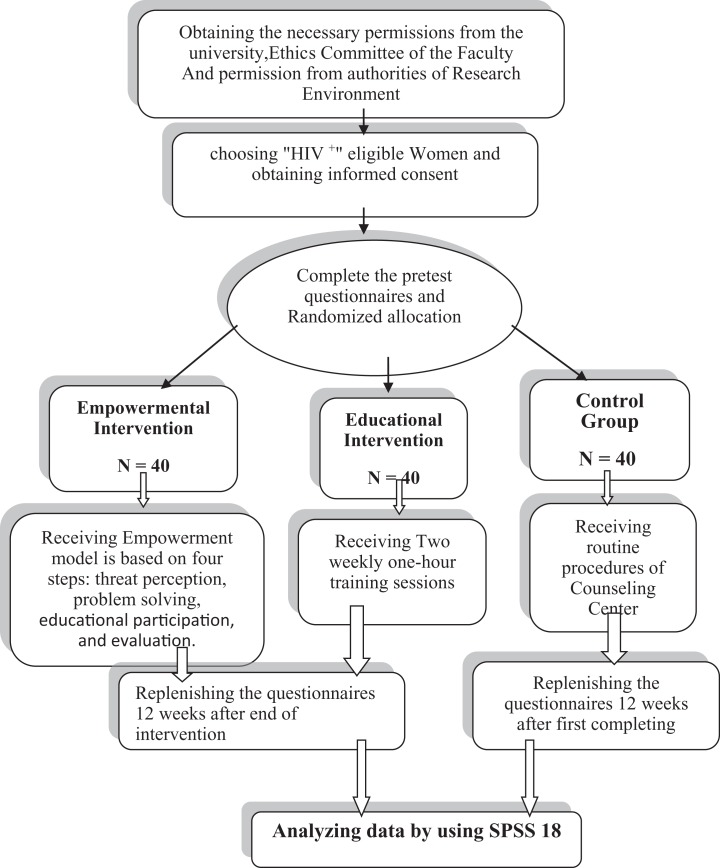
Study methods design.

## Discussion

The present study was conducted to examine in the 3 groups the effect of empowerment and educational interventions on the QOL of women with HIV. The results revealed that the empowerment intervention group experienced an improvement in psychological and spiritual domains and in the QOL. In the educational intervention and empowerment intervention groups, the knowledge assessment scores showed significant differences before and after the intervention, with an increase of scores 12 months after the interventions. It was indicated that the interventions created positive changes in the level of women’s knowledge about the HIV/AIDS.

In this study, the mean age of the women in the control, educational intervention, and empowerment intervention groups was 32.28, 33.68, and 33.38 years, respectively. Given the fact that one factor affecting the QOL was age, the 3 groups were homogeneous in terms of age. In line with our findings, Wang etal showed that 33.9% of HIV-positive women were under 40 years old.^21^ In contrast to the present study’s findings, Gasper et al and Duo Shan et al indicated that 44.3% and 44% of women with HIV were 30 to 39 and 40 to 49 years old, respectively.^11,22^

The majority of the women in the control group (35%), the educational intervention group (35%), and empowerment intervention group (37%) had the high school educational level. This study showed a significant positive relationship between the educational level of the women and their QOL (*P* = .000). Therefore, the educated women enjoyed a better QOL compared to other women. Contrary to our findings, the studies by Gaspar et al and Pereira and Canavarro demonstrated that 41.4% and 46.5% of women had primary and secondary educational levels, respectively.^11,23^ Our findings revealed that a statistically significant relationship was found between husband’s educational level and the women’s QOL. In other words, the husband’s educational level had a positive impact on women’s QOL (*P* = .009).

Our findings highlighted that the majority of the women were housewives: the control (85%), the educational intervention (80%), and empowerment intervention (75%) groups. In a similar study, most women (88%) were housewives and 14% of them were employed.^21^ Another study by Vyavaharkar found out that about three-quarters (77%) of women were unemployed,^24^ and a statistically significant relationship was found between their employment status and QOL (*P* = .002), as employed women had a better QOL than those who were unemployed. In addition, the relationship between husband’s job and all aspects of QOL was statistically significant (*P* = .000).

The majority of the women were infected by HIV through sexual contact: the control (75%), educational intervention (70%), and empowerment (57%) groups. In this regard, Jalalmanesh et al indicated that the most common route of HIV transmission was intravenous drug injection, followed by sexual contact. However, the study by Zimple et al showed that nearly three-quarters of women became infected through sexual contact with heterosexual partners.^16,17^

No statistically significant differences were found in terms of the social relationship, environmental and physical domains, and level of autonomy among the groups. In other words, the empowerment intervention did not affect the QOL of women with HIV.

Findings of Hosseinian et al indicated that the supportive psychotherapy program significantly improved the QOL in the environmental domain (*P* = .001), which was contrary to our study findings. With regard to the autonomy domain, they showed that the supportive psychotherapy program had no impact on the level of autonomy (*P* = .111),^15^ which was in line with our findings. The probable reason for this could be the traditional nature of the society influencing the QOL.

Comparing the psychological and spiritual domains as well as the total QOL in the women, statistically significant differences were found between the groups before and after the intervention. This finding is consistent with the findings of the Howard et al. They found a relationship between the outreach program contacts and retention in care over a 12-month period, especially in those with comorbid mental health or substance abuse conditions and those with multiple barriers to care.^25^ Serena et al also found that outreach programs and interventions could facilitate the engagement of HIV-positive women to care by giving them correct information and increasing their knowledge about the disease.^26^ Judith et al also revealed that an adapted navigation approach referred to as the “HIV system navigation” improved the access to HIV care and guaranteed further development.^27^

Jennifer et al studied a tailored short message service–based intervention for HIV-positive men focused on the reduction of risk-taking behaviors, improvement of HIV knowledge, social support, and patient involvement. They demonstrated a statistically significant increase in HIV knowledge, social support, and the reduction in reported risk behaviors.^28^ Weiss et al conducted a multisite research to develop and implement the effective compounds of behavioral interventions for optimizing the health status of the neglected and understudied population affected by HIV/AIDS. They demonstrated that improving social support, self-efficacy, coping skills, QOL, and the ability of the participants to take an advantage of the necessary health behavior change programs was equally beneficial to less acculturated sections of the affected population.^29^ Our findings regarding the impact of the knowledge and QOL were consistent with ours, and our intervention also was effective in social support. The community must play a much greater role in the provision of HIV treatment, especially for those people who were being left behind.

The United Nations Programme on HIV/AIDS supports organizations that provide health-care services to patients with HIV to ensure all have an equal access to these services. For instance, adherence clubs where patients are given medicines and basic medical checkups are performed, self-formed community antiretroviral therapy groups in which the members distribute medicines to neighbors, and appointment visits where medical visits take place less often and medicines are dispensed for a longer period of time.^4^

Patients with HIV should be recognized as human beings and a humanitarian stance should be taken to find a solution for issues influencing their QOL. Our findings suggested that the empowerment intervention affected the women’s QOL. Therefore, this intervention can be used to increase patients’ knowledge of HIV/AIDS and enhance their psychological and spiritual conditions as well as QOL. Since women are the main pillars of the family structure and the society, interventions aimed at improving their QOL not only improves their survival rate but also strengthens the coherence of the family. Therefore, the provision of education by health-care professionals to women with HIV can be effective in improving their QOL in the society. Also, this study does not have any limitation.
